# The Value of Prenatal First Systolic Blood Pressure Can Predict Severe Preeclampsia and Birth Weight in Patients With Preeclampsia

**DOI:** 10.3389/fmed.2021.771738

**Published:** 2022-02-03

**Authors:** Bei Gan, Xiuyan Wu, Lin Lu, Xuemei Li, Jianhua Li

**Affiliations:** Department of Obstetrics and Gynecology, The First Affiliated Hospital of Fujian Medical University, Fuzhou, China

**Keywords:** preeclampsia, severe preeclampsia, birth weight, systolic blood pressure, prenatal

## Abstract

**Background:**

Preeclampsia is a serious complication of pregnancy that threatens the safety of the fetus and mother. We assessed the relationship between systolic blood pressure (SBP) in the early pregnancy stage (12 weeks) in patients with preeclampsia and the development of severe eclampsia and birth weight.

**Methods:**

Patients were categorized based on the quartiles of the prenatal first SBP level. Logistic regression analysis was performed to assess whether prenatal first SBP was a risk factor for low birth weight and severe preeclampsia. The area under the receiver-operating characteristic curve (AUC) of sensitivity and specificity were used to predict the risk of low birth weight and severe preeclampsia.

**Results:**

A total of 333 patients with preeclampsia were enrolled. There were 162 (48.6%) patients with severe preeclampsia and 270 (81.08%) cesareans. Group I patients with a prenatal first SBP ≤ 119 mmHg prenatal had a higher birth weight. Multiple logistic regression analysis showed that serum creatinine (*p* = 0.025), prenatal first SBP (*p* = 0.029), S-preeclampsia (*p* = 0.003), gestational age (*p* < 0.001), total cholesterol (TC) (*p* < 0.001), and low-density lipoprotein (LDL) (*p* < 0.001) were independent risk factors for low birth weight. Multiple logistic regression analysis showed that prenatal first SBP (*p* = 0.003), TC (*p* = 0.002), and B-type natriuretic peptide (BNP) (*p* < 0.001) were independent risk factors for severe preeclampsia. Compared with Group I (SBP ≤ 119 mmHg), the incidence of low birth weight for patients in groups III (131 ≤ SBP ≤ 138 mmHg) and IV (SBP ≥ 139 mmHg) was significantly higher. Even after correcting for age, gestational age, and biochemical indices, the difference remained statistically significant. The risk of diagnosed severe preeclampsia for patients in Groups IV (SBP ≥ 139 mmHg), III (131 ≤ SBP ≤ 138 mmHg), and II (120 ≤ SBP ≤ 130 mmHg) was significantly higher than that in Group I (SBP ≤ 119 mmHg). The AUC of the prenatal first SBP for predicting low birth weight and severe preeclampsia was 0.676 (95% *CI* 0.618–0.733, *p* < 0.001) and 0.727 (95% *CI* 0.673–0.781, *p* < 0.001), respectively, in patients with preeclampsia.

**Conclusions:**

Prenatal first SBP was associated with birth weight and severe preeclampsia. Higher prenatal first SBP in patients with preeclampsia can predict low birth weight and severe preeclampsia.

## Introduction

Preeclampsia is a serious complication of pregnancy that threatens the safety of the fetus and mother. Preeclampsia and eclampsia, which vary greatly from region to region, cause approximately 50,000 maternal deaths worldwide every year (1). Although the application of magnesium sulfate has reduced the incidence rate of eclampsia (2), it remains an important reason for the threat of maternal life safety. Preeclampsia is mainly characterized by new hypertension and proteinuria and can develop into multiple organ damage, such as visceral, renal, and cerebrovascular damage. Its risk factors mainly include whether the mother has complications, such as chronic kidney disease, hypertension, obesity, familial preeclampsia, previous eclampsia, or fetal intrauterine growth restriction.

Blood pressure is an important indicator for the diagnosis of preeclampsia. Blood pressure higher than 160/110 mmHg is characteristic of the diagnosis of severe preeclampsia (3). Currently, the mechanism of preeclampsia is unclear. Placental ischemia may be one of the important mechanisms leading to preeclampsia, which may occur in the early stages of pregnancy. Hypoxia and energy demands are unbalanced during early pregnancy (4). Studies have reported that a decrease in vasodilation function, circulating nitrous oxide (NO), and an increased cholesterol level are important evidence that endothelial cell injury may precede preeclampsia (5). Currently, there is no relevant report on the relationship between changes in blood pressure in the early stage of pregnancy and the prognosis of patients with preeclampsia. Therefore, this study observed the systolic blood pressure (SBP) at early pregnancy (12 weeks) in patients with preeclampsia and defined the relationship between the SBP level and the development of severe eclampsia and birth weight.

## Methods

### Patients

This study protocol was approved by the ethics committee of the First Affiliated Hospital of Fujian Medical University. All patients provided written informed consent to participate in this study. Patient inclusion criteria included providing written informed consent, aged >18 years, and diagnosed with preeclampsia between June 2015 and July 2021. Patients with infection, trauma, mental illness, hypertension, connective tissue disease, kidney disease, nervous system diseases, multiple pregnancies, congenital anomalies, preterm premature rupture of membrane, diabetes, and those who needed antibiotics, corticosteroids, or immunosuppressive agents within a month were excluded. Of the 362 patients, six were excluded due to connective tissue disease and use of corticosteroids or immunosuppressive agents, eight due to infection or antibiotic use within a month, nine due to chronic kidney disease, and six due to hypertension. Finally, a total of 333 individuals were included.

Clinical data were recorded, including the history of the disease, concomitant disease (kidney disease and hypertension), medications, prenatal first blood pressure, prenatal blood pressure, laboratory data, and delivery model. The primary endpoints were birth weight and severe preeclampsia. Severe preeclampsia was defined by any of the findings of severe features based on the American College of Obstetricians and Gynecologists (ACOG) 2013 criteria: blood pressure ≥ 160/110 mmHg (on two occasions at least 4 h apart), thrombocytopenia (platelet count <100,000/μl), progressive renal insufficiency (serum creatinine ≥ 1.1 mg/dl or doubling serum creatinine in the absence of other renal diseases), new-onset cerebral or visual disturbances, and pulmonary edema (3). Prenatal first blood pressure will be tested at 12 weeks of gestational age. Birth weight <2,500 g was classified as low birth weight and ≥ 2500 g as normal.

### Laboratory Tests

Blood samples were collected before the delivery. Blood biochemical indices, including average values of calcium (Ca^2+^), phosphate(P^3+^), total cholesterol (TC), triglyceride (TG), low-density lipoprotein (LDL) cholesterol, high-density lipoprotein (HDL) cholesterol, albumin (Alb), hemoglobin (Hb), B-type natriuretic peptide (BNP), fibrinogen, uric acid (UA), and lactate dehydrogenase (LDH) were measured in all patients.

### Statistical Analysis

The Kolmogorov–Smirnov test was used to estimate the Gaussian distribution of the data, and *p* > 0.05 indicated a normal distribution. Normally distributed measurement data are represented as mean ± SD, and the Bonferroni test was used for pairwise comparisons among groups. Measurement data that were not normally distributed are represented as median and quartile, and the Mann–Whitney *U*-test was used for comparisons between groups. We categorized patients based on the quartiles of the prenatal first SBP levels within our study population. Comparisons between groups for quantitative data were conducted using the chi-square test or Fisher's exact test. Categorical variables are expressed as numbers (or percentages). A logistic regression analysis was performed to assess whether prenatal first SBP was a risk factor for low birth weight and severe preeclampsia. The area under the receiver-operating characteristic (ROC) curve (AUC) of sensitivity and specificity was used to predict the risk of low birth weight and severe preeclampsia. Statistical significance was set at *p* < 0.05. SPSS 15.0 (SPSS Inc., Chicago, IL, USA) was used for all statistical analyses and figures.

## Results

### Clinical Manifestations and Biochemical Tests

Among the 333 patients with preeclampsia, the mean age was 32.00 ± 5.03 years, and the median birth weight was 2,800.00 (2,100.00, 3,500.00) g. There were 162 (48.6%) cases of severe preeclampsia and 270 (81.08%) cesarean sections. Based on the quartiles of prenatal first SBP, the patients were divided into four groups: group I had a prenatal first SBP ≤ 119 mmHg renatal f; group II had a prenatal first SBP between 120–130 mmHg, group III had a prenatal first SBP between 131 and 138 mmHg, and group IV had a prenatal first SBP ≥ 139 mmHg prenatal. Table 1 shows that the birth weight in groups III and IV was significantly lower than that in group I (*p* < 0.001). The values of Hb, Scr, ACR, and 24 h protein urine were higher in Groups III and IV than that in Group I. Intravenous antihypertensive drugs were used more frequently in groups III and IV. There were no significant differences in LDL, HDL, fibrinogen, and BNP levels between the groups.

**Table 1:** Basic patient demographic characteristics and laboratory data by quartiles of prenatal first systolic blood pressure (SBP) in patients with preeclampsia.

	**All**	**I ≤119 mmHg**	**II 120–130 mmHg**	**III 131–138 mmHg**	**IV ≥139 mmHg**
**General condition**
Number (*n*,%)	333 (100)	84 (25.23)	87 (26.11)	84 (25.23)	78 (23.43)
Age (years, x ± s)	32.00 ± 5.03	30.14 ± 4.27	30.76 ± 4.56	32.96 ± 4.56^**^	34.35 ± 5.64^**^
Gestational age (w)	37.57 (34.29, 39.14)	38.57 (38.14, 39.68)	38.29 (36.14, 39.71)	36.36 (30.46, 38.82)^**^	34.71 (30.14, 37.57)^**^
Cesarean (*n*, %)	270 (81.08)	57 (67.9)	75 (86.21)^**^	66 (78.57)	72 (92.3)^**^
Birth weight [g,M(1/4,3/4)]	2800.00 (2100.00, 3500.00)	3175.00 (2755.00, 3760.00)	3000.00 (2400.00, 3600.00)	2500.00 (1290.00, 3175.00)^**^	2050.00 (1120.00, 2850.00)^**^
S-preeclampsia (*n*,%)	162 (48.6)	15 (17.9)	45 (51.7)^**^	51 (60.7)^**^	51 (65.4)^**^
**Lab tests**
Hb (g/l, x ± s)	122.58 ± 15.27	118.92 ± 13.89	119.62 ± 17.24	128.21 ± 10.95^**^	123.77 ± 16.57^*^
Scr (mmol/l, x ± s)	54.19 ± 14.67	49.85 ± 11.54	53.32 ± 15.22	55.38 ± 12.89^*^	58.10 ± 17.42^**^
UA (mmol/l, x ± s)	317.40 ± 82.22	374.38 ± 82.81	421.83 ± 74.48^**^	422.33 ± 85.78^**^	377.40 ± 84.23
ACR [g/mg,M(1/4,3/4)]	1240.76 (153, 334.14)	453.00 (67.87, 2733.13)	1133.85** (334.47, 1900.10)	1315.36** (101.67–3776.35)	2649.73** (309.96, 4322.81)
24 h protein urine [g/d,M(1/4,3/4)]	1.33 (0.47, 4.03)	0.68 (0.41, 1.82)	1.34** (0.42, 1.83)	1.52** (0.53, 2.84)	4.24** (0.58, 6.50)
Alb (g/l, x ± s)	30.45 ± 4.20	31.70 ± 2.95	31.44 ± 3.43	29.73 ± 5.55^**^	28.87 ± 3.71^**^
TG (mmol/l, x ± s)	3.25 ± 1.32	3.83 ± 1.98	2.76 ± 1.08^**^	3.15 ± 0.95^*^	3.51 ± 1.28
TC (mmol/l, x ± s)	5.94 ± 1.5	6.06 ± 1.76	5.37 ± 1.07^*^	6.06 ± 1.49	6.26 ± 1.60
LDL (mmol/l, x ± s)	3.33 ± 1.22	3.5 ± 1.44	2.99 ± 0.92	3.45 ± 1.21	3.39 ± 1.31
HDL (mmol/l, x ± s)	1.86 ± 0.43	1.75 ± 0.34	1.88 ± 0.48	1.84 ± 0.49	1.93 ± 0.35
Ca (mmol/l, x ± s)	2.03 ± 0.24	2.09 ± 0.25	2.01 ± 0.23^*^	2.03 ± 0.24	1.98 ± 0.21^**^
Fib (g/l, x ± s)	4.65 ± 0.83	4.51 ± 0.77	4.31 ± 0.85	4.29 ± 1.12	4.65 ± 0.83
BNP [pg/ml,M(1/4,3/4)]	54.15 (22.43, 92.19)	56.00 (21.00, 80.00)	64.43 (44.99, 129.93)	32.65 (19.33, 98.01)	48.03 (19.00, 74.78)
LDH (U/L, x ± s)	311.64 ± 156.38	256.46 ± 102.19	327.21 ± 151.16^**^	318.46 ± 169.26^**^	346.35 ± 181.42^**^
GPT (U/L, M1/4,3/4)	17.00 (12.00, 28.00)	16.00 (15.00, 30.00)	16.00 (11.00, 30.00)	21.00 (11.00, 27.25)	15.00 (12.00, 20.00)
**Antihypertensive**
β-blocker (*n*, %)	201 (60.4)	21 (25)	48 (55.2)^**^	60 (71.4)^**^	72 (92.3)^**^
Intravenous (*n*, %)	42 (12.6)	0 (0)	3 (3.4)	21 (25)^**^	18 (23.1)^**^
CCB (*n*, %)	144 (43.2)	21 (25)	33 (37.8)	42 (50)^**^	48 (61.5)^**^

* *Compared with Group I p < 0.05;*

** *compared with group I p < 0.001; Hb, hemoglobin; Alb, serum albumin; Scr, serum creatinine; UA, uric acid; GPT, glutamic pyruvic transaminase; TG, triglyceride; TC, total cholesterol; LDL, low density lipoprotein; HDL, high density lipoprotein; Ca, calcium; BNP, B-type natriuretic peptide; S-preeclampsia, severe preeclampsia; ACR, albumin creatinine ratio; Fib, fibrinogen; LDH, lactate dehydrogenase; Intravenous, intravenous drug application; CCB, calcium blocker*.

### Analysis of Risk Factors for Low Birth Weight and Severe Preeclampsia

Univariate analysis showed that prenatal first SBP was correlated with low birth weight. Multiple logistic regression analysis showed that serum creatinine (hazard ratio [*HR*] = 0.380, 95% *CI* 0.163–0.884, *p* = 0.025), prenatal first SBP (*HR* = 2.652, 95% *CI* 1.107–6.357, *p* = 0.029), S-preeclampsia (*HR* = 4.123, 95% *CI* 1.620–10.493, *p* = 0.003), gestational age (*HR* = 0.043, 95% *CI* 0.017–0.110, *p* < 0.001), TC (*HR* = 0.093, 95% *CI* 0.026–0.329, *p* < 0.001), and LDL (*HR* = 22.816, 95% *CI* 6.208–88.859, *p* < 0.001) were independent risk factors for low birth weight (Table 2).

**Table 2:** Logistic regression analysis risk factors for low birth weight in patients with preeclampsia.

	**Univariate analysis**		**Multi variate analysis**	
	**HR**	***P***	**HR**	***P***
Age (32 years-old)	3.048 (0.538–17.271)	0.208		
Hb (123 g/l)	2.693 (0.504–14.395)	0.247		
Scr (54.88 umol/l)	0.198 (0.033–0.970)	0.002	0.380 (0.163–0.884)	0.025
UA (391 umol/l)	0.154 (0.017–1.423)	0.099		
Alb (30.2 g/l)	0.029 (0.002–0.382)	0.007		
TG (3.41 mmol/l)	0.046 (0.006–0.383)	0.004		
TC (6.07 mmol/l)	0.037 (0.004–0.329)	0.003	0.093 (0.026–0.329)	<0.001
LDL (3.35 mmol/l)	37.766 (4.890–291.664)	<0.001	22.816 (6.208–83.859)	<0.001
HDL (1.89 mmol/l)	6.803 (0.961–48.177)	0.055		
Ca (1.92 mmol/l)	5.442 (0.939–31.518)	0.059		
Fibrinogen (4.4 g/l)	0.161 (0.025–1.023)	0.530		
BNP (54.3 pg/ml)	1.690 (0.365–7.823)	0.502		
24 h protein urine (1.66 g/d)	0.972 (0.298–3.167)	0.962		
Prenatal first SBP (130 mmHg)	5.793 (1.556–21.562)	0.009	2.652 (1.107, 6.357)	0.029
S–preeclampsia	2.400 (1.570–12.608)	0.005	4.123 (1.620–10.493)	0.003
Gestational age (35.7 weeks)	0.038 (0.028–0.243)	<0.001	0.043 (0.017–0.110)	<0.001

*HR, hazard ratio; Hb, hemoglobin; Alb, serum albumin; Scr, serum creatinine; UA, uric acid; TG, triglyceride; TC, total cholesterol; LDL, low density lipoprotein; HDL, high density lipoprotein; Ca, calcium; BNP, B-type natriuretic peptide; and S-preeclampsia, severe preeclampsia*.

Univariate analysis showed that the first prenatal SBP was correlated with severe preeclampsia. Multinomial logistic regression analysis showed that prenatal first SBP (*HR* = 3.490, 95% *CI* 1.539–7.915, *p* = 0.003), TC (*HR* = 3.582, 95% *CI* 1.599–8.020, *p* = 0.002), BNP (*HR* = 9.167, 95% *CI* 3.737–22.486, *p* < 0.001) were independent risk factors for severe preeclampsia (Table 3).

**Table 3:** Logistic regression analysis risk factors for severe preeclampsia in patients with preeclampsia.

	**Univariate analysis**		**Multi variate analysis**	
	**HR**	***P***	**HR**	***P***
Age (32 years-old)	8.273 (2.204–31.057)	0.002		
Hb (123 g/l)	0.159 (0.042–0.598)	0.007		
Scr (54.88 umol/l)	0.11.932 (0.555–6.722)	1.932		
UA (391 umol/l)	0.173 (0.047–0.642)	0.009		
Alb (30.2 g/l)	2.487 (0.645–9.585)	0.186		
TG (3.41 mmol/l)	1.084 (0.328–3.580)	0.895		
TC (6.07 mmol/l)	5.674 (1.849–17.407)	0.002	3.582 (1.599–8.020)	0.002
LDL (3.35 mmol/l)	0.698 (0.128–3.809)	0.678		
HDL (1.89 mmol/l)	0.381 (0.078–1.874)	0.381		
Ca (1.92 mmol/l)	1.944 (0.601–6.287)	0.267		
Fibrinogen (4.4 g/l)	1.221 (0.342–5.223)	0.329		
BNP (54.3 pg/ml)	53.297 (8.836–321.485)	<0.001	9.167 (3.737–22.486)	<0.001
24 h protein urine (1.66 g/d)	4.097 (1.700–9.876)	0.002		
Prenatal first SBP (130 mmHg)	10.542 (2.705–41.090)	0.001	3.490 (1.539–7.915)	0.003
Birth weight	1.148 (0.304–4.337)	0.839		
Gestational age (35.7 weeks)	3.715 (0.835–16.520)	0.085		

*HR, hazard ratio; Hb, hemoglobin; Alb, serum albumin; Scr, serum creatinine; UA, uric acid; TG, triglyceride; TC, cholesterol; LDL, low density lipoprotein; HDL, high density lipoprotein; Ca, calcium; and BNP, B–type natriuretic peptide*.

### Relationship Between Prenatal First SBP and Low Fetal Weight, Severe Preeclampsia

Compared with patients in group I (≤ 119 mmHg), the risk of low birth weight for patients in group III (120–130 mmHg) and IV (≥ 139 mmHg) were significantly higher. After correcting for age, gestational age, and biochemical indices, the difference remained statistically significant. For patients in group IV (≥ 139 mmHg statistic), the risk of low birth weight significantly increased by 271.8% compared with that in Group I (≤ 119 mmHg hat in Gr) (*p* < 0.001) (Table 4).

**Table 4:** Association of prenatal first SBP and low birth weight.

	**I ≤119 mmHg**	**II 120–130 mmHg**	**III 131–138 mmHg**	**IV ≥139 mmHg**
n with low birth weight/total	18/84	39/87	45/84	48/78
Unadjusted	1	1.970 (1.058–3.669) *P =* 0.033	2.436 (1.301–4.561) *P =* 0.005	5.730 (2.908–11.291) *P < * 0.001
Fully adjusted^a^	1	2.604 (1.160, 2.805) *P =* 0.107	2.534 (1.309–4.905) *P =* 0.006	3.718 (1.779–7.771) *P < * 0.001

a *Adjusted for age, Hb, Scr, Alb, TC, LDL, 24 h protein urine, and gestational age*.

Among the patients who were diagnosed with severe preeclampsia, the risk of severe preeclampsia diagnosed in Group IV (≥ 139 mmHg), III (120–130 mmHg), and II (120–130 mmHg) was significantly higher than that in Group I (≤ 119 mmHg); this difference remained statistically significant even after correcting for all potential confounding factors (Table 5).

**Table 5:** Association of prenatal first SBP and severe preeclampsia.

	**I ≤119 mmHg**	**II 120–130 mmHg**	**III 131–138 mmHg**	**IV ≥139 mmHg**
n with s–preeclampsia /total	15/84	45/87	51/84	51/78
Unadjusted	1	4.929 (2.45, 9.915) *P < * 0.001	7.109 (3.497–14.454) *P < * 0.001	8.689 (4.197, 17.987) *P < * 0.001
Fully adjusted^a^	1	5.978 (2.133, 16.765) *P =* 0.001	6.125 (2.514, 14.919) *P < * 0.001	5.109 (1.971, 13.503) *P < * 0.001

a *Adjusted for age, Hb, Scr, Alb, TC, LDL, 24 h protein urine, and gestational age*.

### ROC Analysis Prenatal First SBP Predicting Low Birth Weight and Severe Preeclampsia

**Figure 1:**
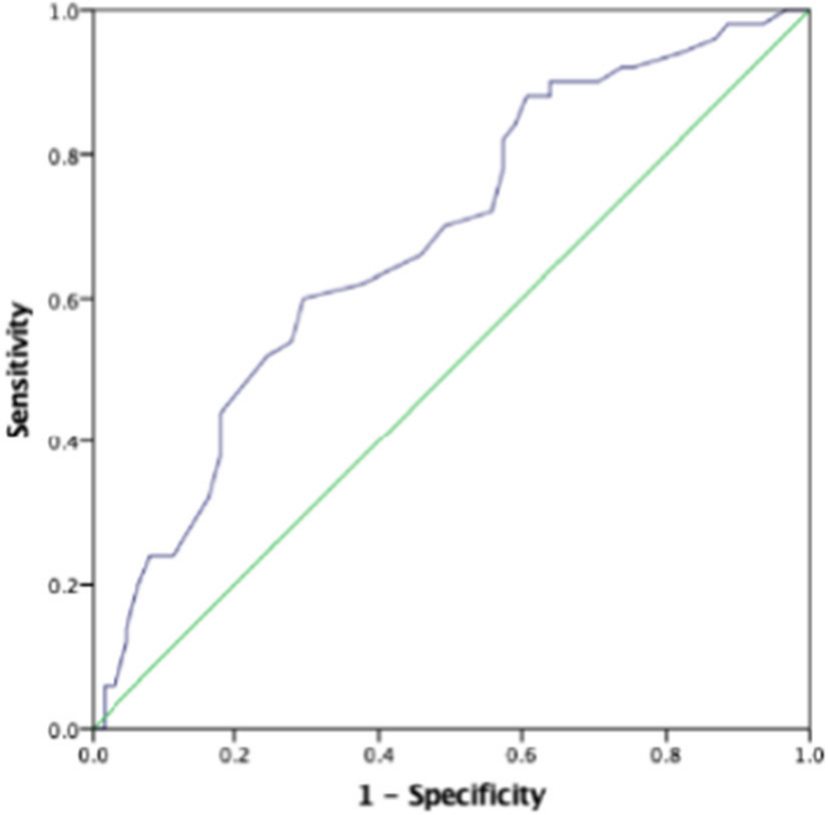
The role of prenatal first systolic blood pressure (SBP) in predicting low birth weight. The receiver operating characteristic (ROC) curve illustrates prenatal first SBP. Areas under the curves (AUCs) are 0.676 (95% *CI* 0.618–0.733, *p* < 0.001) for the prenatal first SBP. A cut-off valuable of 121.5 mmHg yielded good sensitivity and specificity. The sensitivity and specificity are 88 and 60.7%, respectively.

**Figure 2:**
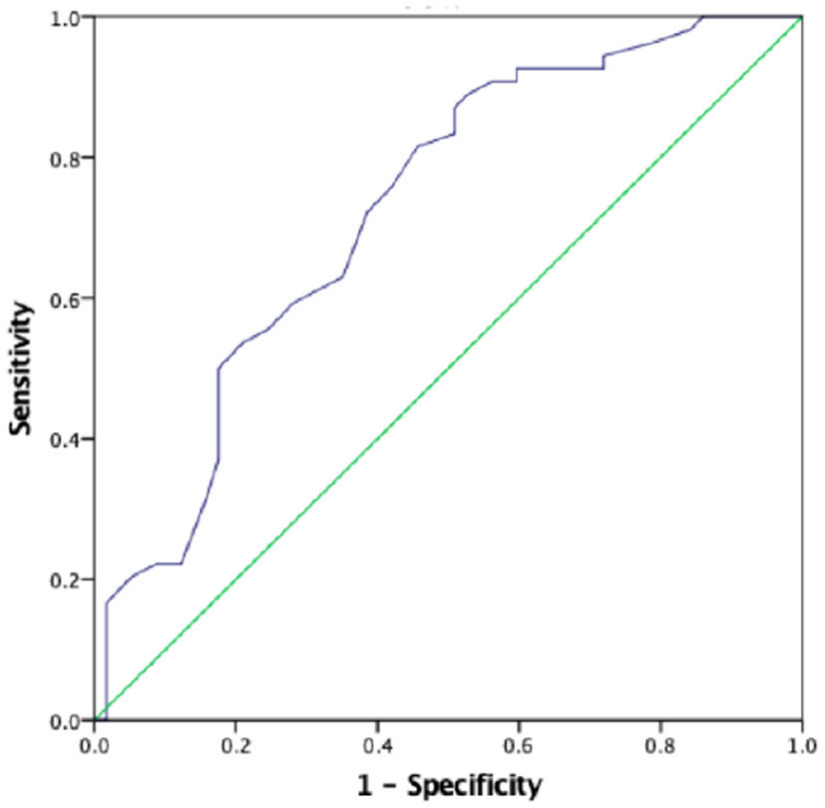
The role of prenatal first SBP in predicting severe preeclampsia. The ROC curve illustrates prenatal first SBP. AUCs are 0.727 (95% *CI* 0.673–0.781, *p* < 0.001) for the prenatal first SBP. A cut-off valuable of 123.0 mmHg yielded good sensitivity and specificity. The sensitivity and specificity are 88.9 and 52.6%, respectively.

The AUC for the first prenatal SBP to predict low birth weight and severe preeclampsia was 0.676 (95% *CI* 0.618–0.733, *p* < 0.001) and 0.727 (95% *CI* 0.673–0.781, *p* < 0.001), respectively, in patients with preeclampsia, which showed that prenatal first SBP had a high accuracy for predicting low birth weight and severe preeclampsia. A cut-off value of 121.5 and 123 mmHg yielded good sensitivity and specificity for predicting low birth weight (88 and 60.7%, respectively) and severe preeclampsia (88.9 and 52.6%, respectively) (Figures 1, 2).

## Discussion

Preeclampsia and eclampsia seriously threaten the safety of pregnant mothers and fetuses. The incidence rate of hypertensive disorders in pregnancy, such as preeclampsia and eclampsia, pregnancy with hypertension, and chronic hypertension, is 5.5% (6). Patients with preeclampsia or eclampsia have a 3–25-fold increased risk of serious complications during pregnancy, such as pulmonary edema, disseminated intravascular coagulation, placental abruption, preterm birth, and aspiration pneumonia. Preeclampsia risk factors include family history, genetic susceptibility, maternal smoking, pregnancy, maternal age, *in vitro* fertilization, and maternal physical conditions, such as hypertension, diabetes, chronic kidney disease, and obesity.

The ACOG divides gestational hypertension into four categories: preeclampsia and eclampsia, chronic hypertension, chronic hypertension combined with preeclampsia, and gestational hypertension. In this study, patients with preeclampsia had no history of chronic hypertension before pregnancy, and their blood pressure was monitored and recorded at the first follow-up of prenatal examination at 12 weeks. At 12 weeks, even if the blood pressure was within the normal range, the proportion of patients with severe preeclampsia and low birth weight was significantly higher in the SBP ≥ 120 mmHg group than in the SBP ≤ 119 mmHg group. The risk of low birth weight was 2.604–3.718 times and severe preeclampsia was 5.109–6.125 times. These abnormalities in women with preeclampsia suggest that the mechanisms that contribute to placental ischemia are set into motion very early in pregnancy. Thus, the concept of defective placentation and failure to transform uterine spiral arteries has emerged to be central to the pathogenesis of preeclampsia (7, 8).

Endothelial dysfunction may be an important cause of high blood pressure. Studies have shown that the imbalance of circulating angiogenic factors plays an important role in the etiology of maternal syndrome (9–11). High levels of anti-angiogenic factor soluble fms-like tyrosine kinase1 (sFLT1) produced in the placenta and released into the maternal circulation led to the dysfunction of maternal endothelial cells, leading to preeclampsia (12–16). sFLT1 is a soluble splice variant of the membrane-bound receptor vascular endothelial growth factor receptor 1 (VEGFR1) that binds to the proangiogenic proteins VEGF and placental growth factor (PIGF) and mediates angiogenic signaling *via* cell surface receptors (10, 17). Patients with higher levels of sFLT1 may have more serious diseases and worse clinical prognoses (15, 18–20). NO is an effective vasodilator that mediates the effects of PIGF and VEGF *in vitro* (21–23). The circulating NO level of women with preeclampsia decreases may be relatively insufficient in the early stages of pregnancy (24–26). Hypertension in patients with preeclampsia is not mediated by the renin-angiotensin-aldosterone system. However, it may be mediated by antiangiogenic factors and agonistic autoantibodies that bind to the angiotensin II type 1 receptor (AT1-AAs) (21, 27). This antibody may exist in women with early preeclampsia (28), leading to an increased risk of cardiovascular events in preeclampsia, thus progressing to severe preeclampsia. In animal experiments, AT1-AAs in pregnant mice can upregulate sFLT1 and induce fetal growth restriction (29). In this study, among the patients with preeclampsia in the early stage of pregnancy, the birth weight in the group with an SBP ≥ 131 mmHg was significantly lower than that in the group with an SBP ≤ 119 mmHg, and AT1-AAs may appear in the early stage of pregnancy and lead to fetal growth retardation. Therefore, in the early stage of pregnancy in patients with preeclampsia, even if the blood pressure of the patient is within the normal range, patients with lower SBP have a higher birth weight, longer gestational weeks, and lower probability of severe preeclampsia.

Gestational age is closely related to birth weight. A study shows that patients with preeclampsia can predict birth weight and heavily influence perinatal survival. With the advancing gestational age, the different impacts on birth weight between patients with preeclampsia and healthy pregnant women become small (30). In this study, gestational age in patients with preeclampsia was close to the birth age. Logistic regression analysis showed that gestational age was an independent risk factor for low birth weight. In addition, we found that SBP in the early stages of pregnancy has a relationship with gestational age. The lower the systolic blood pressure in the early-stage pregnancy, the longer the gestational age they have. Therefore, monitoring the blood pressure in the early stages of pregnancy may predict the gestational age and low birth weight in patients with preeclampsia.

This study has some limitations. First, because AT1-AAs can upregulate the sFLT1 gene and induce maternal endothelial cell damage and fetal growth retardation, the levels of AT1-AAs and sFLT1 were not detected in the early stage of pregnancy to further prove the relationship between SBP and preeclampsia. Second, the population included in this study was patients with preeclampsia, and there were no healthy mothers as controls. It is not clear whether SBP in the early stages of pregnancy can predict adverse outcomes, such as preeclampsia. Finally, this was a retrospective observational study. The corrected confounding factors were limited to the current recognized and measured data. Therefore, we need to follow up with these patients to observe whether SBP in early pregnancy can predict maternal prognosis and complications.

In conclusion, high SBP in the early stages of pregnancy in patients with preeclampsia is closely related to the occurrence of severe preeclampsia and low birth weight. Even after adjusting for risk factors, SBP in the early stage of pregnancy is an independent risk factor for predicting the occurrence of severe preeclampsia and low birth weight. Closely monitoring pregnant women with high SBP in early pregnancy may reduce the incidence of severe preeclampsia, reduce the incidence of newborns with low birth weight, and improve the survival rate of newborns.

## Data Availability Statement

The raw data supporting the conclusions of this article will be made available by the authors, without undue reservation.

## Ethics Statement

The studies involving human participants were reviewed and approved by Ethics Committee on human of the First Affiliated Hospital of Fujian Medical University. The patients/participants provided their written informed consent to participate in this study.

## Author Contributions

BG wrote the manuscript, conceived the study, and participated in its design. XW made the statistics and enrolled the patients. JL and LL planed and supervised the study. XW and XL collected and enrolled patients. All authors read and approved the final manuscript.

## Funding

This study was supported by Correlation between sFlt-1/PIGF ratio and fetal intrauterine growth restriction (Education and Scientific Research Project for middle-aged and young teachers in Fujian Province: JAT160189), Correlation among FABp4, PTEN, and gestational diabetes mellitus (Fujian Science and Technology Planning Project, 2017Y0034), and the Obstetrics and Gynaecology Division of the First Affiliated Hospital of Fujian Medical University.

## Conflict of Interest

The authors declare that the research was conducted in the absence of any commercial or financial relationships that could be construed as a potential conflict of interest.

## Publisher's Note

All claims expressed in this article are solely those of the authors and do not necessarily represent those of their affiliated organizations, or those of the publisher, the editors and the reviewers. Any product that may be evaluated in this article, or claim that may be made by its manufacturer, is not guaranteed or endorsed by the publisher.
